# A role for contractile vacuole and papilla in transition from cyst to telotroch in *Vorticella microstoma*

**DOI:** 10.1098/rsos.250894

**Published:** 2026-06-17

**Authors:** Eric Galiana, Marie-Line Kuhn, Agnès Attard

**Affiliations:** ^1^ Université Côte d'Azur, INRAE, ISA, Sophia Antipolis 06903, France

**Keywords:** ciliate, *Vorticella*, cyst, telotroch differentiation, basal–apical polarity

## Abstract

Unicellular ciliates survive adverse environments by forming resting cysts. Using video microscopy and quantitative imaging, we established that differentiation of swimming telotrochs and their release from a cyst followed a strict temporal and spatial pattern in *Vorticella microstoma*. The temporal pattern was initially marked by formation of a contractile vacuole, which discharged its fluid contents into a newly formed cytopharynx and a membrane invagination. With the pulsatile rhythm of the vacuole, the membrane invagination differentiates into an oral cavity that exhibits polarized cilia. The spatial pattern was delineated by two poles: (i) an apical pole, defined by the position of the pre-existing papilla at the cyst wall, determined the site of oral cavity formation; (ii) a basal pole at the point of rupture of the cyst wall allowed telotroch release. Both temporal and spatial patterns were impaired by concanamycin A, an inhibitor of vacuolar-type H^+^-ATPases that alters contractile vacuole functions. Our findings indicate that the transition from cyst to telotroch is predetermined by the papilla location at the cyst wall and is controlled by the contractile vacuole. The *Vorticella* cyst provides a simple, single-cell model for investigating basic principles of integrated organelle neogenesis in protozoa and apical–basal cell polarity in eukaryotes.

## 1. Introduction

Van Leeuwenhoek [1] observed the first microbes, presumably *Vorticella*, with his light-refracting lens microscope. He described them as ‘poor little creatures’ living in rainwater, having a round or oval body, moving or harpooning themselves towards the extremity, where they possess a contractile tail (stalk), and clumping together in a few filaments. The *Vorticella* genus now comprises more than 100 different sessile peritrichous ciliates [2]. They evolve in marine, freshwater and terrestrial environments, either moving or attached to various substrates such as plant/animal surfaces and appendages, biofilms and flooded objects [3–5]. They have been characterized as dominant protozoans, putatively playing a role in fertilization on nitrogen supply to the rhizosphere soil during the initial growth of *Beta vulgaris* L. [6], and as potential biocontrol agents against plant-parasitic nematodes [7].

At the cellular level, *Vorticella* develop in at least three single-cell forms: free-swimming telotrochs; sessile, stalked trophonts; and cysts, the last ones being formed under adverse environmental conditions such as a lack of nutrients or oxygen, drought, osmotic stress and extreme temperatures [8–10]. The free-swimming telotroch has generally an inverted bell-shaped morphology with a basal part exhibiting a row of cilia ensuring movement. The apical part is surrounded by at least two rings of cilia emerging from the peristome, which surrounds the open oral cavity or mouth. Immediately behind is a cytopharynx, which results from a plasma membrane invagination, connecting together the oral cavity, particularly, to the food vacuoles (the digestive organelles) and to the single contractile vacuole (CV). The osmoregulatory CV is presumed to empty its contents into the cell’s cytopharyngeal region through an elongated tube [11]. A small spherical micronucleus and a large bean-shaped macronucleus are also present, as well as the other eukaryotic organelles. The sessile trophont form has the same morphology and exhibits a retractile stalk (100–150 µm) that attaches the cell to a surface. The vortex water flow generated by cilia beating around the oral cavity draws bacteria food particles towards the peristome and thence to food vacuoles that ingest particles by phagocytosis. *Vorticella* species, with their trophont-stalk retracting almost instantly (10–20 mm s^−1^) and the high swimming speed (0.2–1 mm s^−1^) of the free telotroch, are among the fastest cellular ‘micromachines’, making them highly attractive models for studying movements from the hydrodynamic, cellular and molecular point of views [10,12–14].

Perhaps because cyst formation is a slower process, the dynamics of encystment and excystment (telotroch release from the cyst) are less well documented [15]. Cyst formation results in the formation of an envelope, the cyst wall, encapsulating a diffuse cytoplasm/hyaloplasm, with indistinct cilia, peristome, cytopharynx and vacuoles. Under favourable conditions, excystment occurs, primarily regulated by osmolarity and characterized by cyst differentiation, functional cilia and CV [9].

In protists, osmoregulation is achieved by the CV, which maintains osmotic and ion homeostasis [16–19]. The cycle of CVs involves three sequential events: diastole (slow extension of the vacuole as it gradually fills with excess water drained from the endoplasm); systole (rapid vacuolar contraction); and expulsion (the release of vacuolar fluid from the CV out of the cell). This process is referred to as ‘beating’. To maintain osmotic and ion homeostasis through extrusion of water and excess of ions, the CV may exploit the H^+^-gradient generated by the organelle-resident vacuolar ATPases (V-type H^+^-ATPase; V-ATPase) [16,17,20]. The V-ATPase is highly conserved in eukaryotic organisms and is assembled into two structural multimeric protein complexes: a transmembrane V0-proton pump and a cytoplasmic V1-ATPase that can be reversibly detached and reattached [21,22]. In protists such as *Dictyostelium* and *Phytophthora* [23,24], the function of vacuolar contraction is impaired by concanamycin A, a specific inhibitor of V-ATPases that binds to the V0 subunit c [25].

To date and for a long time ago, knowledge of the *Vorticella* cyst still remains meagre [15]. The underlying machinery controlling encystment and excystment remains unexplored since the first study by Finley and Lewis [9]. Here, we combine quantitative imaging with drug-inhibited V-ATPase activity to characterize the dynamics of excystment and telotroch release from the *Vorticella microstoma* cyst induced in response to hypo-osmotic conditions. We established that before release, oral cavity and cilia neogenesis, as well as cyst wall deformation followed the differentiation of the CV and that their formation was tightly synchronized both spatially and temporally with the acceleration of the pulsatile rhythm of the diastole-systole-expulsion sequence in the CV. We showed that inhibition of V-ATPase-mediated proton pumping was associated with very significant deficits in telotroch release, speed of motion, as well as cilia organization and distribution. We propose that the *Vorticella* cyst is a simple model for investigating basic principles of integrated neogenesis of organelles in protozoans, and of basal–apical cell organization in eukaryotes.

## 2. Material and methods

### 2.1. *Vorticella* strain and sample treatment

The *V. microstoma* 30 897 strain, in co-culture with *Enterobacter aerogenes* (13 048 strain), was purchased from the American Type Culture Collection collection of protists (LGC standards). The cells were cultured for 3–14 days in V8 liquid medium at 24°C. Three days were necessary to achieve a high proportion of swimming telotrochs. A culture period of 10–14 days was required to reach a 100% rate of cyst formation. Between these times, the trophont stage was most commonly observed. Excystment was induced by hypo-osmotic stress: medium from 10–14-day-old cultures was discarded, and cysts were resuspended in distilled water (time point *t* = 0). Samples (100–200 µl) were rapidly mounted in a chamber made from a microscope slide and a top coverslip resting on two spacers (0.5–1 mm) for immediate observation.

The function of the CV was tested as previously described for *Dictyostelium discoideum* [23]. Concanamycin A, which binds to the proteolipid subunit c of the V0 complex, was used to inhibit V-ATPases [25]. Cysts in the stationary phase were harvested by centrifugation (3000*g*), resuspended in distilled water to create a hypo-osmotic environment and treated or not with 5 µM concanamycin A in solution (dimethyl sulfoxide (DMSO)). The appropriate DMSO dilution without the drug was applied to the control samples.

### 2.2. Microscopy

Microscopy was performed using a Zeiss Axio Z1 microscope with a plan-apochromat 63×/1.40 oil M27 and an EC-plan-neofluofluar 40×/0.75 M27 objective. Images (size 512 × 512 or 1029 × 1044 pixels) and movies (image 512 × 512 pixels; time scale 0.2 or 0.4 s; time series 1000–3000 s) were collected with a Hamamatsu camera using [12] software (Carl Zeiss Microscopy, Germany).

### 2.3 Immunohistochemistry

Cysts and telotrochs were collected by centrifugation. They were fixed in methanol for 10 min at −20°C and then incubated in phosphate-buffered saline (PBS), pH 7.2 at room temperature (RT). Samples in PBS were treated with 0.1% Triton for 20 min at RT, and later with a blocking solution containing 3% of bovine serum albumin in PBS, for 30 min. Primary antibody incubations (18 h at 4°C) consisted of a rabbit anti-α-tubulin subunit polyclonal antibody (NeoBiotech), an anti-V1-ATPase B1 subunit (NeoBiotech) or anti-β-actin diluted 1 : 200. Samples were washed three times with PBS and incubated for 1 h at RT with a FluoProbes 547H donkey anti-rabbit IgG or a Fluoprobes 488 anti-rabbit IgG antibody, diluted 1 : 200 (Interbiotech—BioScience Innovations). Samples were rinsed three times and mounted in Fluoroshield with DAPI (Sigma, St. Louis, MA USA).

### 2.4. Image time series analysis

Kymography was used to analyse beating of the CV, cytopharynx and oral cavity expansion using the open source FIJI [26] and the Measurement_Tool plugin (k**-**button; https://dev.mri.cnrs.fr/projects/imagej-macros/wiki/Velocity_Measurement_Tool). Selected segmented lines, including a diameter and the centre of the vacuole, or delimiting as much as possible the mid-section of the papilla, oral cavity, cytopharynx and CV, were used for generating kymograms. Space was expressed on the *x*-axis and time on the *y*-axis. To determine mean velocity of cysts and telotrochs, cells were tracked with FIJI using the TrackMate plugin [27] as per the procedure detailed by Lupatelli *et al.* [14].

### 2.5. Statistics

Experiments were repeated at least three times. Data are presented as mean values ± s.e.m. One-way ANOVA with Mann–Whitney pairwise test was used for statistical analysis with the PAST software [28], and data are presented as mean ± s.e.m. Bonferroni-corrected/adjusted *p*-values of <0.05 were considered significant.

## 3. Results

### 3.1. Excystment main features

Encystment and excystment have been reported in several ciliated protists [29,30], in a few species within the *Vorticella* genus [15], including *V. microstoma* [9], which was investigated in the present study. Encystment was induced in 100% of cells after incubation of *V. microstoma* cells in V8 medium for 10–14 days. At these time points of incubation, no trophont nor telotroch was detectable with light microscopy. The dynamics of encystment was not further examined in this study, which focused on excystment. To study the dynamic of excystment, this cyst population was exposed to hypo-osmotic shock. In these conditions, the rate of excystment was estimated at between 30% and 50% within 1–2 h. At this time, microscopy revealed that all released cells were telotrochs, either free-swimming or immobile near the cyst wall from which they had been released. Telotrochs that were not yet swimming and were almost exclusively located immediately adjacent to the empty cyst wall from which each had emerged were investigated. In the results presented below, the timing of each event is expressed in relation to the moment (*t* = 0) when the osmotic stress was applied. A labelled diagram of the main structures observed in a cyst and a telotroch is shown in figure 1A.

**Figure 1.**
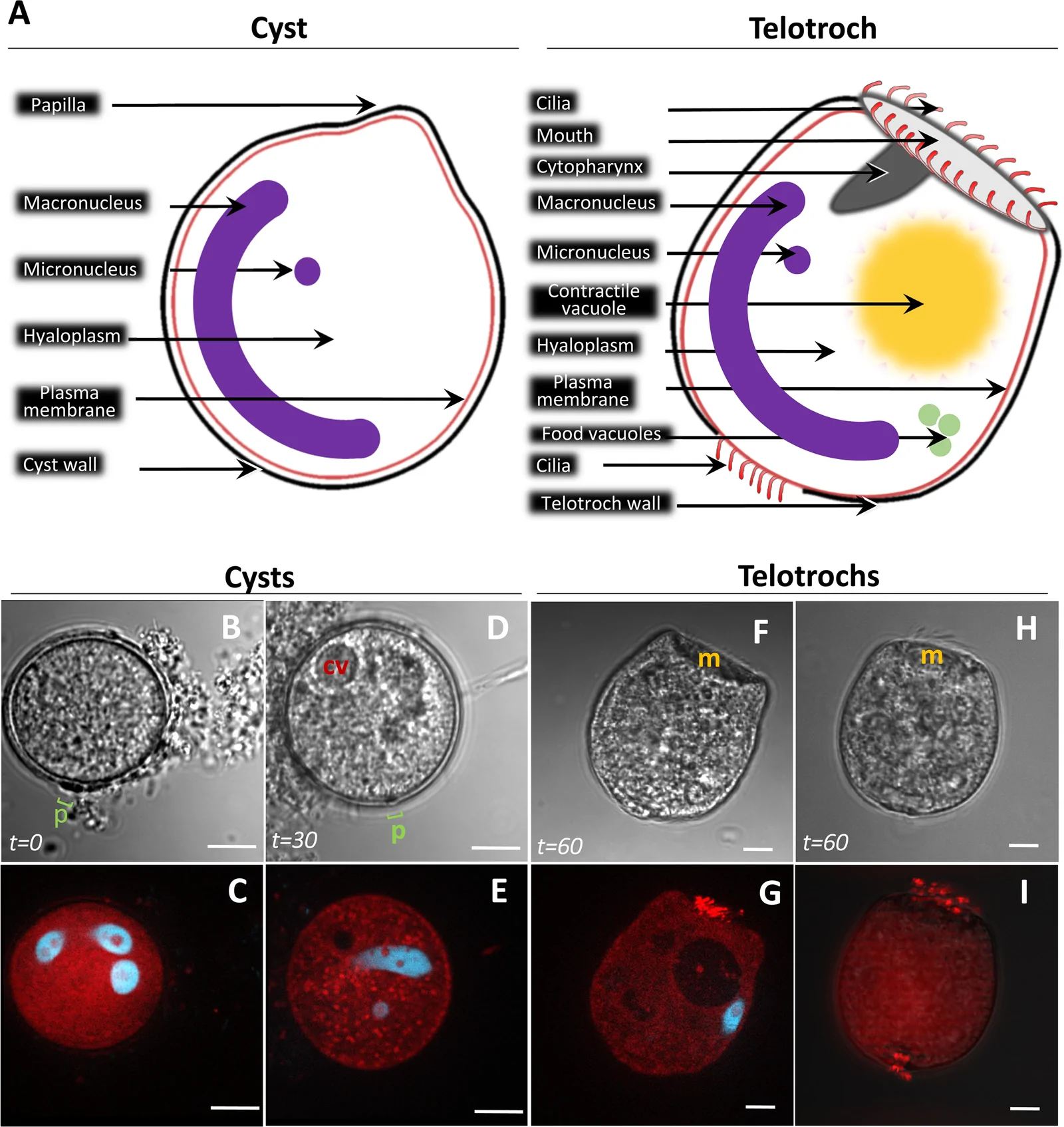
Cyst and telotroch morphologies. (A) Schematic representation of cyst and swimming telotroch showing the main subcellular structures in *Vorticella*. (**B, D, F, **H) Representative images of phase-contrast micrographs 0, 30 and 60 min after induction of the hypo-osmotic stress: cysts (**B*,***D), telotrochs (**F*,***H). (**C, E, G,**I) Representative images of immunofluorescent staining for α-tubulin (red) and DAPI of polylobar nuclei (blue) in cysts (**C*,***E) and telotrochs (**G, **I). In the optical section presented in panel I, micronucleus or macronucleus structures could not be detected by the DAPI staining. Different structures are annotated as followed: (*p*) papilla, (cv) CV and (*m*) oral mouth/oral cavity. Scale bar: 10 µm.

Prior to the stress, all *Vorticella* material consisted of perfectly circular and encapsulated cysts (diameter: 28.8 ± 0.9 µm) with a papilla located at one pole (figure 1B,D). Immunostaining for the α-tubulin subunit revealed an intracellular and diffuse staining, homogeneously distributed in the cytoplasm, suggesting that microtubules were either not present or were poorly polymerized and that cilia were resorbed (figure 1C). At approximately *t* = 30 min post-stress, differentiation was initiated, as attested by the formation of the CV (figure 1D) and positive staining for α-tubulin in the cytoplasm, which became punctate, suggesting αβ-tubulin oligomerization into clusters (figure 1E). At *t* = 30–60 min, numerous telotrochs were released from cysts. Cells exhibited the characteristic telotroch bell morphology, decorated with cilia (figure 1F,H). Their size (1595.5 ± 58.8 µm^2^) was increased by 2.4 times, compared with cysts (571.2 ± 36.9 µm^2^) (*p* = 0.0005; *n* = 14). Immunostaining for α-tubulin (figure 1G,I) clearly indicated microtubule polymerization and cilia neogenesis at the apical pole (the oral cavity) of the telotroch (figure 1G,I) and at its basal pole (figure 1I). When visible in the field, the rupture point of the cyst wall at release was located opposite to the papilla (electronic supplementary material, figure S1). This suggested that at the time of rupture, pressure varied along a particular cellular axis. In support of this hypothesis, the release of the telotroch systematically occurred according to its apical–basal polarity with the basal end engaging first (electronic supplementary material, figure S1).

### 3.2. Tracking neogenesis of early organelles

To further examine the spatio-temporal dynamics of organelle neogenesis within the cyst (CV, oral cavity, cytopharynx, cilia, cyst wall deformation), samples were placed in a microscope chamber slide under hypo-osmotic stress. Cysts and excystment were captured at five frames per second, until telotroch release (figure 2; electronic supplementary material, videos S1–S3). Events captured in sequential images were processed by kymography and are summarized in figure 2.

**Figure 2.**
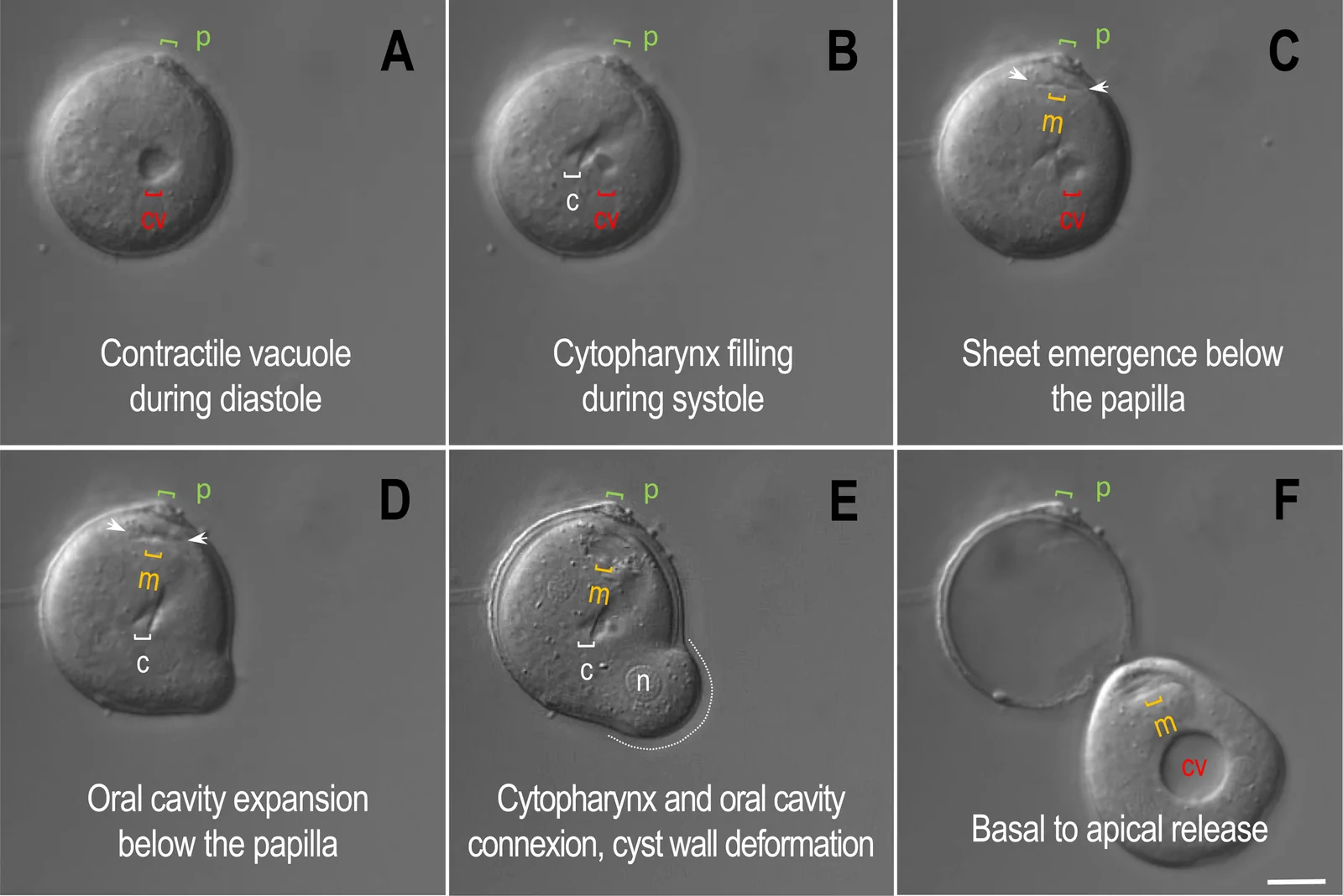
Time-lapse imaging of excystment. (**A**–F) A representative sequence of events observed once cells were already in hypo-osmotic conditions and cyst differentiation had commenced (extract from electronic supplementary material, videos S1). The location of various structures is indicated as follows: (p) papilla; (cv) contractile vacuole; (c) cytopharynx; (m) oral mouth/oral cavity and (n) nucleus. The dotted white line indicates the location of cyst wall deformation. The micrographs show the sequential steps in cyst differentiation: (A) the CV during diastole; (B) the cytopharynx filling during systole; (C) a thin sheet emerging beneath the papilla, as indicated by the two white arrows showing its position; (D) the oral cavity expansion from the thin sheet; (E) the cytopharynx-oral cavity connection, and the cyst wall deformation; (F) basal to apical release. Scale bar: 10 µm.

For the CV, we retrieved from kymograms the number of pulsations before release, kinetics of beating frequency and size of the vacuole at the end of systole (figure 3). The data show that the timing of the first pulse was variable and occurred quite a long time (7–21 min range) after stress induction (figure 3A,B). The total number of beats before release was constant (41 ± 4, figure 3C). Contractile frequency ranged between 0.01 and 0.05 Hz, and the distribution over time revealed the same pattern in all samples: first slow beats with a progressive increase in frequency followed by a decrease for the last 5–10 beats before release occurred (figure 3D; electronic supplementary material, figure S2). These variations were mainly due to variability in the duration of diastole, while the duration of each systole was short and constant, lasting approximately 0.8–1 s. As shown in figure 3A, the CV increased in size over time, reaching its maximal volume just before release. The overall size was not measured by kymographic data because the position of the vacuole in relation to the line selected along the diameter changed marginally due to the effect of low flow or Brownian motion acting on the cyst. Changes in CV diameter were quantified manually at three points: at the first (C_f_) and last (C_l_) beat detected within the cyst, and then within the telotroch immediately after release (T) (figure 3E). At C_l_, the diameter was 1.9 ± 0.5-fold larger than at C_f_ and 1.6 ± 0.4-fold larger at T than at C_l_. Thus, it was estimated that during differentiation from cyst to telotroch, the volume of the CV increased approximately 15–20-fold over time.

**Figure 3.**
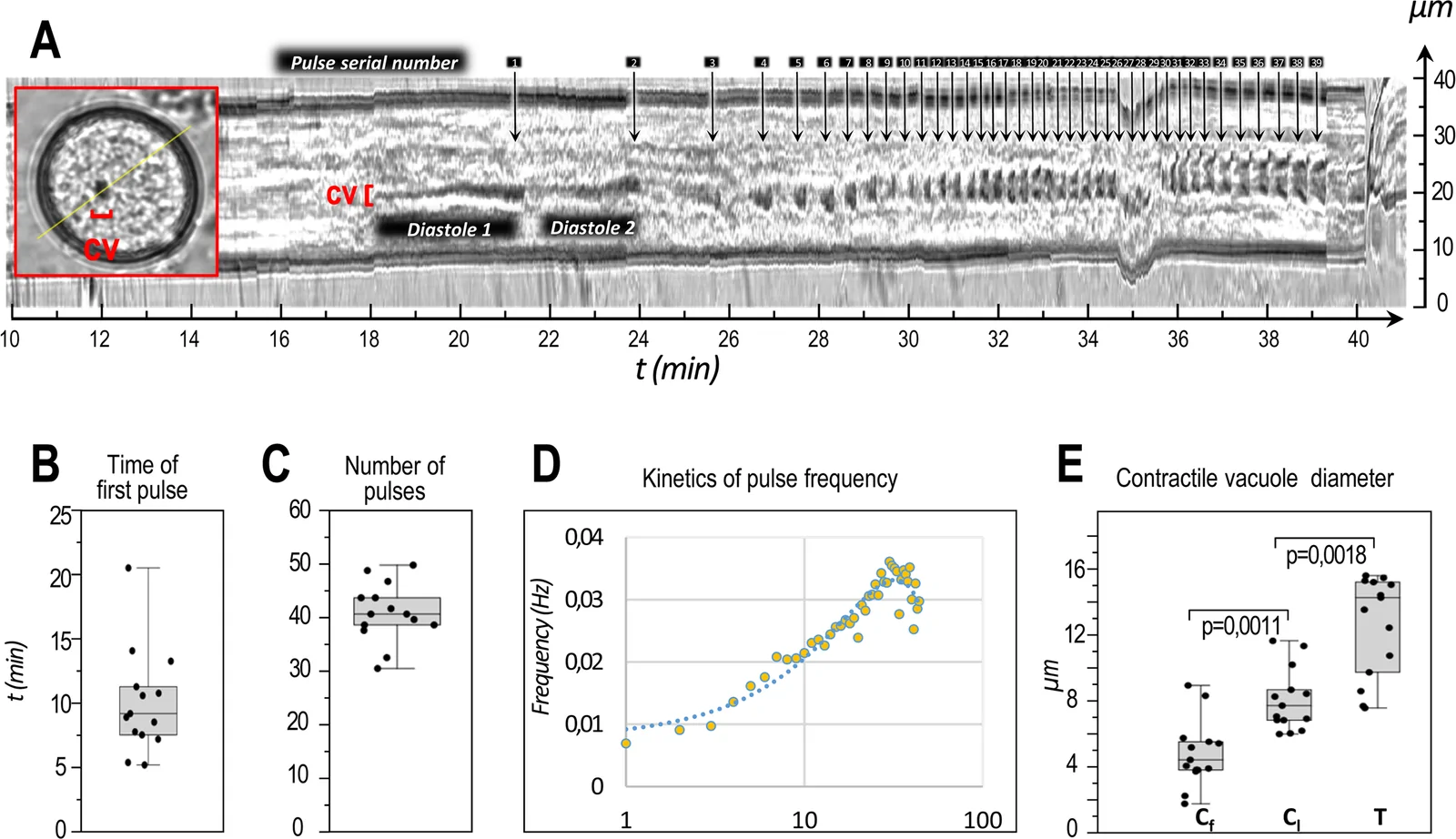
Kymogram of differentiation from cyst to telotroch. (A) Kymogram generated from electronic supplementary material, video S2 showing the kinetics of CV beats. Inset shows the position of the section-line selected and the localization of the CV during the first detected diastole. On the kymogram are indicated: (i) the position of CV, (ii) the duration of the first and second diastole detected, and (iii) vertical arrows indicate the end of each diastole in serial beats. (B) Boxplot of time for detection of a first pulse (*n* = 14). (C) Boxplot of number of pulses counted from *t* = 0 to telotroch release (*n* = 14). (D) Mean CV contraction frequencies in each time series of beats (*n* = 9). (E) Boxplot of vacuole diameter: within the cyst at the first (C_f_) and last (C_l_) beat detected, and within telotroch (T) at release (*n* = 14).

The timing of neogenesis of the cytopharynx was more difficult to determine. However, when cytopharygeal structures first became visible in several successive time lapse sequences, they clearly appeared to correlate with the ability of the CV to contract (electronic supplementary material, videos S1–S3). Moreover, some or all of the CV fluid discharged rapidly into the cytopharynx at systole. Indeed, the cytopharyngeal volume began to increase at the exact moment when systole started (marked by a decrease in vacuole volume) and coincided with the end of diastole (figure 2A,B; figure 4). The maximal increase in volume of the cytopharynx matched the same time as systole duration 0.8–1.2s (figure 4A–D) notwithstanding the frequency of image acquisition, which limited further temporal resolution of these two rapid events.

**Figure 4.**
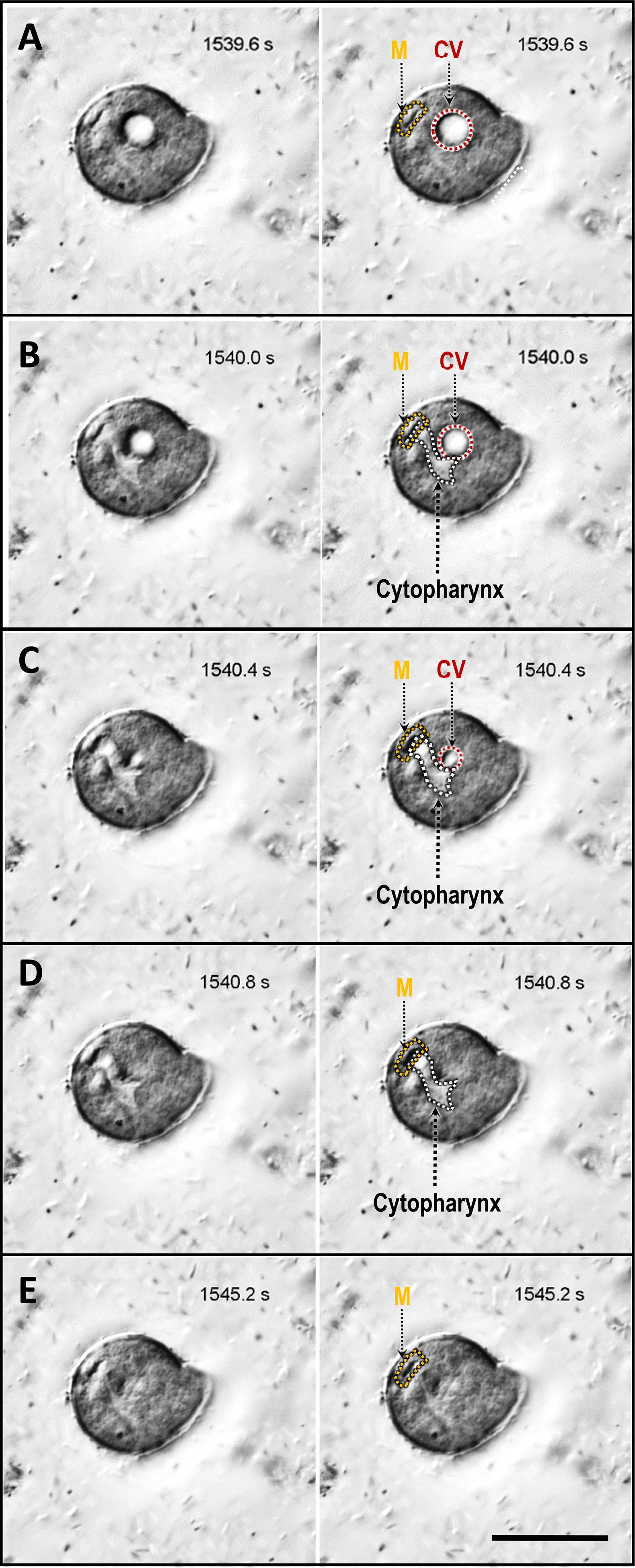
Time-lapse imaging of systole and cytopharynx filling. (**A**–D) Static images in duplicate, without (left) and with annotation (right), and taken in electronic supplementary material, video S3 at 0.4 s intervals. Images show the proximal continuity between oral cavity (M), cytopharynx and contractile vacuole (CV) location during the last systole before telotroch release. (E) taken immediately prior to a new diastole. Scale bar, 30 μm*.*

Remarkably, refined temporal analysis also revealed that the oral cavity arose from a thin sheet, perhaps an invagination of the plasma membrane, the expansion of which was cadenced by the rhythmic contraction of the CV (electronic supplementary material, videos S1–S3). This primitive thin sheet was first visible within the hyaloplasm, always directly beneath the papilla (figure 2C; electronic supplementary material, video S1), probably involving a specialized cell cortex area for anchorage. At this stage, neither formed nor functional cilia were visible. The sheet gradually segregated by rhythmic expansion, evolving into a cavity under successive repetitions of CV beats. It is proposed that the CV may empty its fluid contents into the cytopharyngeal region that links to the oral cavity by forming a tube (electronic supplementary material, video S1). It is also noteworthy that when a kymogram was drawn based on a selected line crossing organelles from the papilla to the vacuole (figure 5A,B), the oral cavity enlargement timely and perfectly coincided with the transition from diastole to systole (figure 5C). The progressive enlargement was only detected at the late stages of differentiation. At these stages, the emergence of cilia was detected (figure 2E,F), and vibration of the cilia was perceptible as the cavity expanded just before and after release (electronic supplementary material, videos S1–S3). Once formed, this cavity constituted the oral cavity, appearing to remain closed throughout the cyst differentiation–telotroch differentiation sequence.

**Figure 5.**
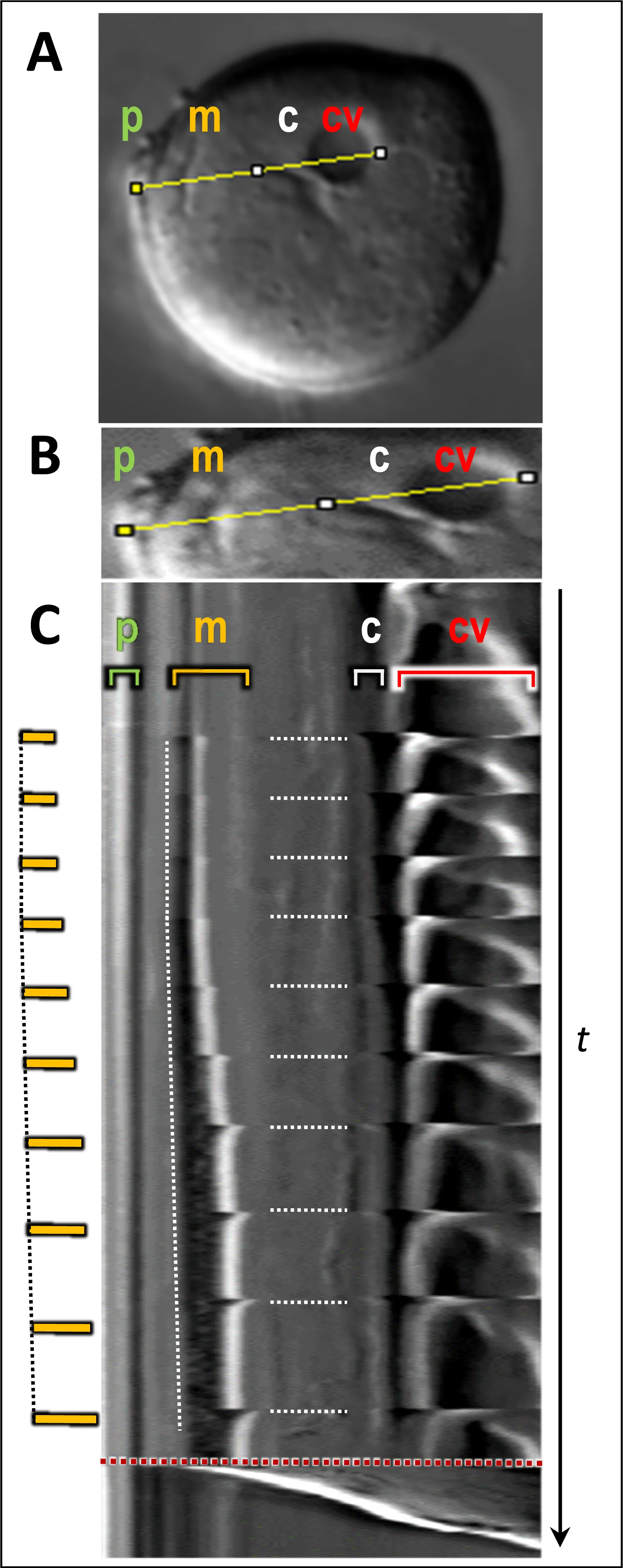
CV regulation of the rate of enlargement of the developing oral cavity. (**A, **B) images indicating how the section line was positioned for kymogram generation. The section line crosses the papilla (p), oral cavity (m), cytopharynx (c) and CV (cv). (C) Kymogram of the last eleven beats of the CV before telotroch release. Horizontal dotted white bars highlight the synchronization of CV systole, filling of the cytopharynx and progressive rhythmic oral cavity enlargement. Bold yellow bars highlight the incremental opening of the oral cavity in rhythm with the last CV beats before telotroch release (indicated by the red dotted line).

### 3.3. Deformation and rupture of the cyst wall

In the last stages of cyst differentiation, initial deformation of the cyst wall occurred at various time over a prolonged interval (10–200 s) before the telotroch was released (figure 6A). Analysis of sequential images revealed that once initiated plastic deformation of the cyst wall occurred continuously during the period prior to rupture. Final rupture was abrupt (less than 0.2 s), faster than the resolution of the video image capture and hence was not quantified.

**Figure 6.**
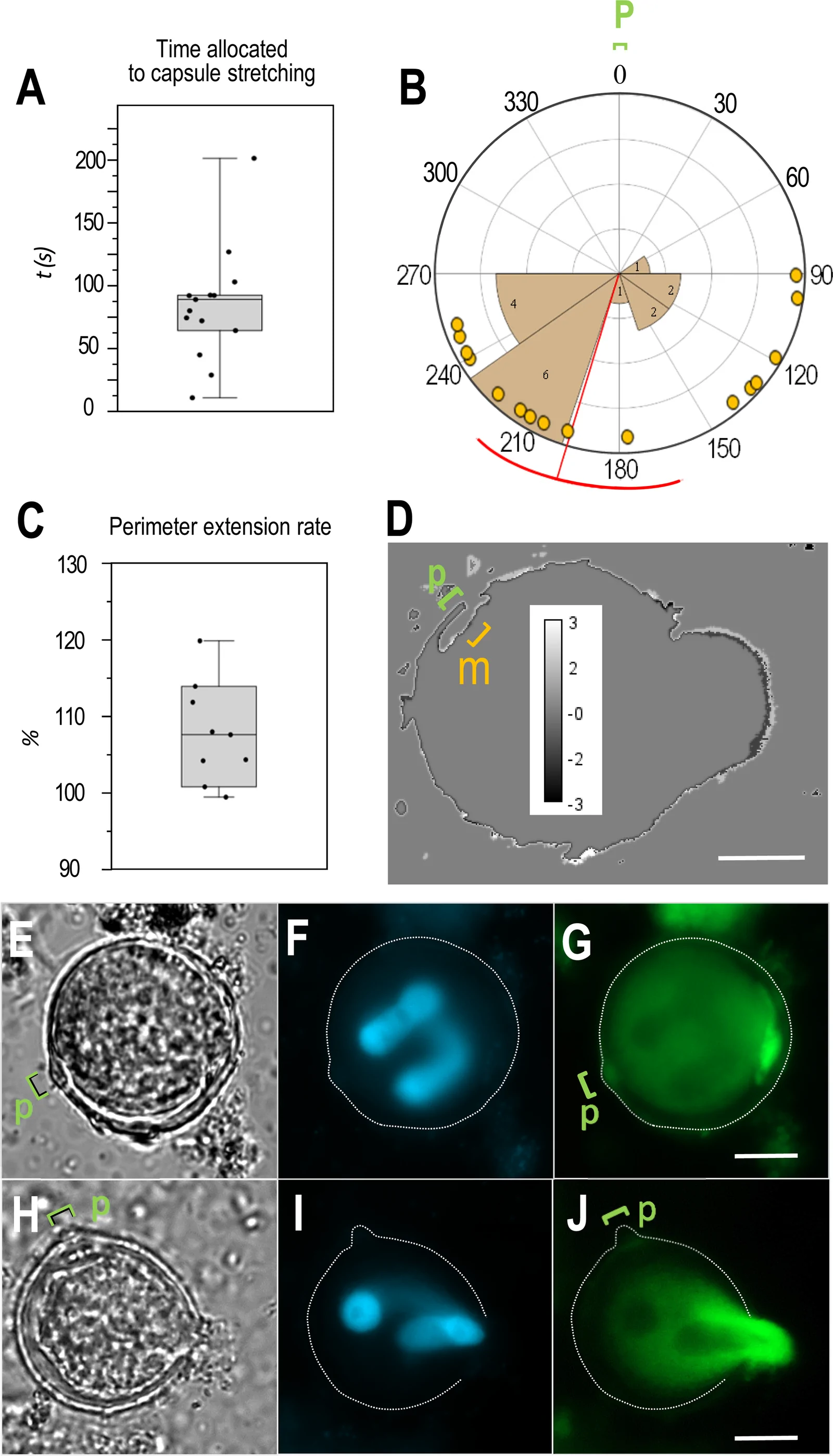
Deformation and rupture of the cyst wall. (A) Time of cyst wall stretching from protrusion emergence to telotroch release (*n* = 14). (B) Polar plot of angle between the position of the papilla and the rupture point: range of angles between the line passing through the midpoint of the papilla (p; *θ* = 0°) and the centre of of the cyst mass, and the half-line passing through the centre of the cyst mass and the midpoint defined at the site of protrusion where plastic deformation occurs and subsequently telotroch release. This range only contains obtuse angle values (*n* = 16). (C) Percentage of increase in cyst size calculated as the ratio of the perimeter measured just prior to release to the perimeter before protrusion (*n* = 8). (D) Curvature of a cyst perimeter just prior to release, showing local deformation of the cyst wall, the location of the papilla (p) and oral cavity (m). Calibration bar indicates value for point curvature (µm^−1^). (E–J) Phase contrast (**E*,*** H), DAPI staining of polylobar nuclei (F*,* I) and actin immunostaining (G*,* J) of cysts during release. Scale bar, 10 μm*.*

Plastic deformation resulted from a protrusion of the leading edge taking place in an angular position located in the semicircle opposite to the papilla coordinates (figure 6B). This became the rupture point for the subsequent telotroch release (electronic supplementary material, figure S1). The plastic deformation resulted in an increase in the perimeter of the cyst (over time, figure 6C) to 24.2 ± 7.9 µm immediately prior to release (figure 6D), illustrating the significant elastic/elongation capacity of polymers in the cyst wall. Deformation and rupture of the cyst wall were associated with local actin accumulation revealed by immunostaining just before (figure 6E–G) or during release (figure 6H,I), suggesting that protrusion at the leading edge requires actin recruitment.

### 3.4. Concanamycin A inhibits cyst differentiation and telotroch release

To initiate exploration of machinery controlling excystment, we assessed the function of the CV complex, the first organelle that could be observed before others such as cytopharynx and oral mouth. The investigation was focused on the V-ATPases that generate H^+^-gradients across membranes and are abundant in the CV membranes of different protozoa living in a wide range of osmotic environments. They are important for vacuolar function in expelling the water that enters cells by osmosis [11]. Immunostaining of cysts and telotroch with an antibody against the B1-subunit of the V1-ATPase subcomplex showed localization of the V1-ATPase subcomplex in the plasma membrane and CV membrane of *Vorticella,* as seen for other protists (figure 7A–D). The typical crescent-shaped staining of a single vacuole among the food vacuoles and the contractile one suggested localization was specific to the CV.

**Figure 7.**
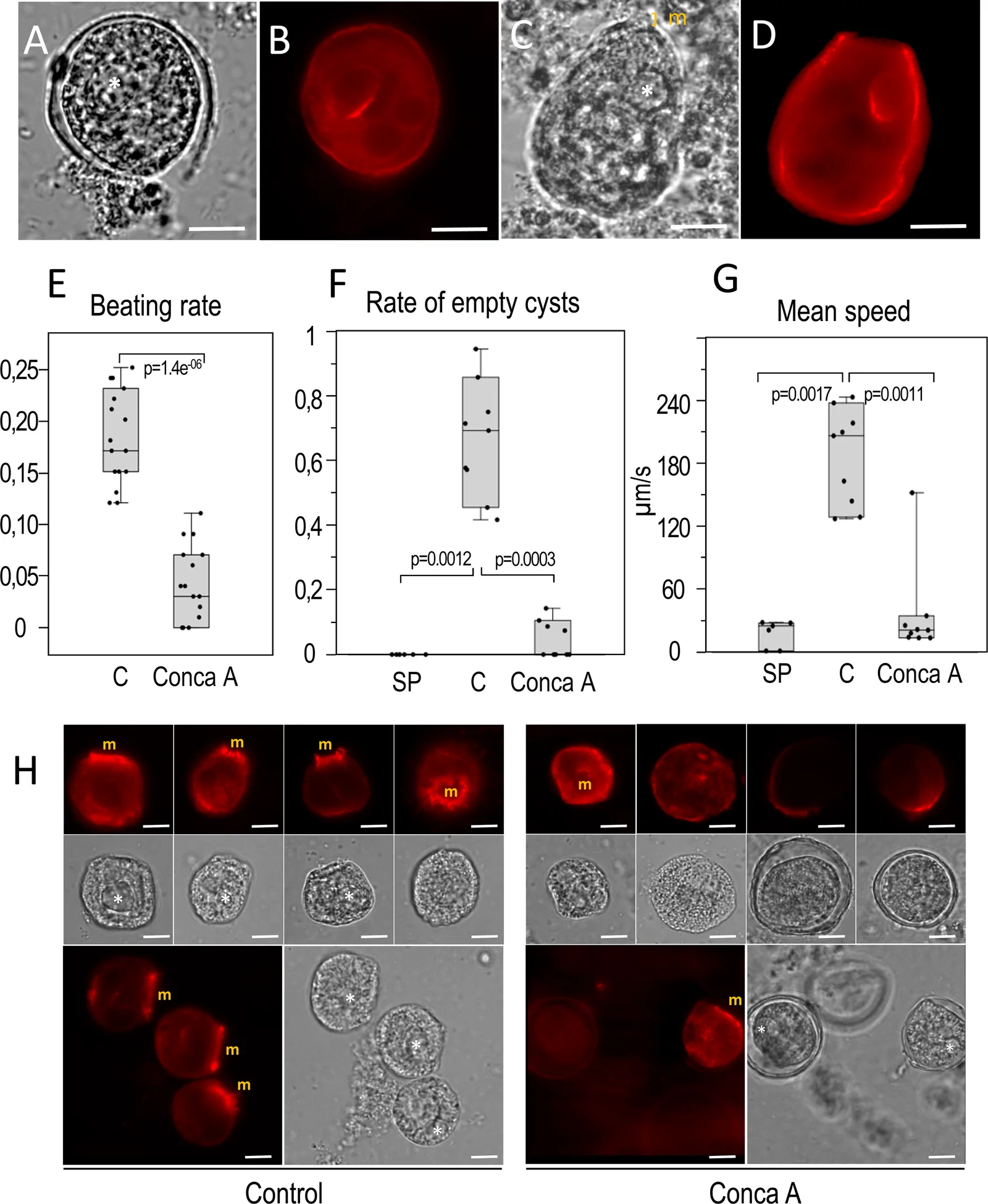
Concanamycin inhibits differentiation and release. (A–D) Phase contrast microscopy (**A*,***C) and immunostaining for the B1-subunit of the V1-ATPase subcomplex (**B*,***D) of a cyst (**A*,***B) and a telotroch (**C*,***D). (E) Rate of cysts with a CV beating once during a 20 s sequence and after 30 min of incubation in the presence of concanamycin A (Conca A) or absence (control; C). (F) Proportion of empty cysts and (G) telotroch mean speed, at the stationary phase (SP), after 1 h of release in water in the presence of concanamycin A (Conca A) or absence (control; C). (H) Phase contrast microscopy and immunostaining for α-tubulin of representative cysts and telotrochs treated or not with concanamycin A. White asterisks indicate the CV, and ‘m’ indicates the oral cavity position. Scale bar, 10 μm*.*

Next, we examined if the concanamycin A, a specific inhibitor of the V-ATPases, impaired the CV in *Vorticella*. Cysts in the stationary phase were collected, washed and then incubated in water in the presence or not of 5 µM concanamycin A. At 30 min, we calculated the rate between cysts exhibiting a CV beating at least one time and all cysts observed on sixteen 20 s sequences. A reduced rate (4.2 instead of 18.3) attested that concanamycin A impaired the ability of CV to beat (figure 7E). At 60 min, we also counted filled versus empty cyst walls to calculate the ratio between empty and total cysts numbers (figure 7F). As expected, no empty cyst wall was detected in cysts at the stationary phase (SP; 14 days culture in V8 medium), while a high rate of empty cyst walls was calculated in the control (C) after 1 h of incubation in water. In concanamycin A-treated samples (Conca A), the rate of empty cysts was drastically reduced compared with control cells (figure 7F). Concomitantly, there was also a marked decrease in the mean speed of moving cells in the presence of the drug (figure 7G), suggesting that cysts were also impaired in the differentiation process affecting the motion apparatus. To evaluate if the motion apparatus was properly formed, we visualized α-tubulin by immunostaining. In the control cells, the α-tubulins were mainly distributed at the two poles of most released telotrochs (figure 7H) drawing the characteristics two ciliary arrays of *Vorticella* sp., which promote fluid flow for cell motility. In the concanamycin A-treated cells, cysts and telotrochs were faintly stained compared with the control cells (figure 7H; electronic supplementary material, figure S3A, B). The telotrochs could exhibited a staining more uniformly distributed and not reminiscent to a polarized status. This indicated that ciliogenesis, involving polarized basal body docking onto cell membranes, was inhibited or slowed (figure 7G). Thus, concanamycin A altered CV beating, inhibited telotroch release and cyst differentiation.

## 4. Discussion

Paradoxically, although it was probably the first microorganism described in detail [1], *Vorticella* sp*.* remains ‘poor little creatures’, being among the most neglected organisms in terms of available data in both cellular and molecular biology and genomics. Only 329 nucleic acid and 9 protein sequences are accessible in databases such as NCBI or Uniprot. It also seems to have not yet appeared in the Protist 10 000 Genomes Project [31]. However, its distinctive single-celled forms (cyst, telotroch, trophont) make it highly suitable for investigation of the mechanisms that govern biological transitions within a species. Using microscopy and pharmacology, the present study explored the cyst–telotroch transition enabling excystment in the ciliate *V. microstoma*.

### 4.1. The cyst excystment

Li *et al.* [30] reviewed a vast amount of information available on cellular changes and known mechanisms involved during the excystment process in 14 representative ciliated protists. The general principles by which excystment is induced involved firstly the reappearance of ciliary and intracellular structures that had degenerated during encystment and before excystment could occur. Secondly, escape from the cyst wall occurs via a preformed or newly formed pore on the cyst wall and rupture generated by the pressure of cell movement inside the cyst wall and excystment vacuole. In *Pleurotricha lanceolate,* generation of the pore did not occur at a predetermined position [32].

In the current study, data indicated that the papilla and the CV orchestrate a complete differentiation of a telotroch from a cyst initially constituted of unstructured hyaloplasm. Two patterns characterized the cyst transition to telotroch. The first pattern is temporal, initially marked by the functional formation of the CV, which discharges its fluid into the newly formed cytopharynx and thence into a membrane invagination, which, to the beating rhythm of the CV, differentiates into an oral cavity. The second pattern is spatial, delineated at one pole by the position of the papilla, which determines the site of the oral cavity formation and ciliogenesis directly below it, also dictated by the CV tempo. The point rupture of the cyst wall, which allows the release of the telotroch in an apical–basal direction, defines the second pole and is characterized by actin accumulation. These data emphasize the role of CV and papilla in the two patterns setting up (figure 8).

**Figure 8.**
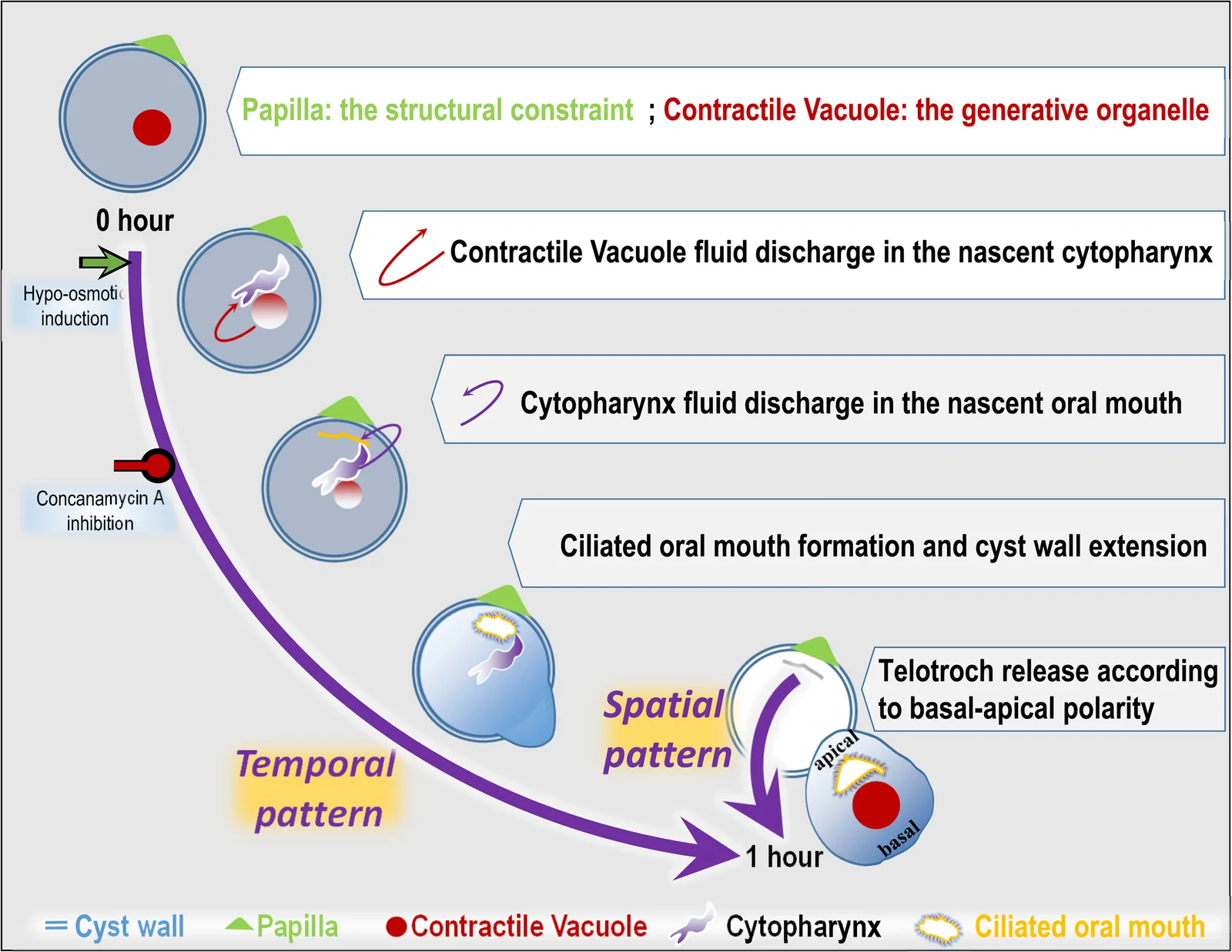
Events in the formation and release of a telotroch from a cyst in *Vorticella microstoma.* Step-by-step schematic representation of the proposed cyst-telotroch based on data in the present study: coordination within the cyst of the papilla position at the cyst wall and a functional CV, induces formation and orientation of organelles (cytopharynx and ciliated oral cavity) as well as apical–basal telotroch polarity, and subsequent telotroch release.

Indeed, two lines of evidence designate the CV as being if not the conductor of the first temporal pattern, at least as a key upstream player. (i) It is the first functional organelle to develop, and under the strict tempo of its contractions, the cytopharynx appears, followed by the oral cavity. (ii) Treatment with concanamycin A, to inhibit V-ATPase-dependent functions in the CV, altered ciliogenesis, polarized ciliary arrangement, release and motion of telotrochs. CV could act by discharging signalling molecules to the plasma membrane of nascent organelles (cytopharynx, oral cavity), as *D. discoideum* CV discharges cAMP and Ca^2+^ to the plasma membrane at the rear of each cell during early cell aggregation, allowing stalk formation [33]. CV could also contribute to excystment by regulating osmotic and ion homeostasis, consistent with the increase in the size of released telotrochs. A possible mechanism is that the turgor pressure generated by the telotroph size increase stretched the cyst wall to its maximum capacity up to a breaking point. Considering the actin enrichment at the site of the cyst wall break (figure 6G,J), a complementary or alternative mechanism is that actin assembly pushing against the cyst wall causes a deformation and break.

A key, though probably passive, player in the spatial pattern definition is clearly the papilla. Its position defined where in the plasma membrane the oral cavity would emerge, precisely under the papilla. In this way, it also determined the apical–basal polarity of the telotroch. Considering that telotroch release always occurred according to its basal–apical polarity and from within the semicircle opposite to the papilla, papillae can be considered as the organizers of the spatial pattern of excystment. Thus, the position of the papilla may be regarded as a focal point for a developmental process and not simply a morphological ornament significant in taxonomy.

### 4.2. Morphogenesis of apical–basal polarity

More broadly, telotroch differentiation in *Vorticella* may serve as a new model in which to investigate the apical–basal polarity that is essential to many morphogenetic processes in eukaryotes. Cellular polarity has been extensively studied at the genetic and molecular level in higher eukaryotes [34], in ciliates [35] or amoebozoa as the unicellular *D. discoideum* [33]. In *Vorticella,* the establishment of apical–basal polarity was achieved within the cyst before release, resulting from a single-cell process primarily involving the same two structures: the generative CV and the positional papilla. The pre-existing papilla of the cyst wall served as a structural constraint, defining the position of the apical organization of future telotrochs through attachment at a particular cortical area of the plasma membrane from which the oral cavity emerged. This finding supports the hypothesis of ‘structural inheritance’, whereby pre-existing structures in ciliates enforce a developmental plan for newly formed structures [36]. In *Tetrahymena pyriformis* and *Paramecium tetraurelia*, the pre-existing intracellular cortical organization of cilia provides the plan for the formation and positioning of new structures (basal bodies, microtubular network), thus defining the cortical organization after scission [35–37]. It is also reminiscent of maternal tissues imposing mechanical constraint in higher eukaryotes [38] such as the maternal cell wall-mediated control of apical–basal patterning in brown algae [39,40]. The present data support the papilla in *Vorticella*, being the ‘extracellular’ anchorage point for cellular material assembled during encystment and it being required for oral cavity and cilia array formation during excystment. Its composition may be fibrillary, such as that seen after release in figure 2F and electronic supplementary material, video S1, still linked to the papilla site after release, and consistent with the shape of the thin sheet formed beneath the papilla (figure 2C). The establishment of apical–basal polarity also required a functional generative CV complex. The analyses of the movies clearly showed the CV expelling fluid through the pore in the cytopharynx. The discharge was then mainly propelled to the thin sheet located at the papilla, apparently contributing to oral cavity and cilia array formation. The discharge wave did not propagate to the basal region of the cell below the vacuole. Thus, fluid discharge appears to define a developmental apical pathway from the CV to the papilla where cytopharynx and oral cavity genesis can take place.

### 4.3. Opening up

Encystment and excystment are fundamental to the conservation and dissemination of protists in the biosphere. Most of the cellular and molecular mechanisms remain unknown. We have highlighted generative and structural elements arising from a cyst and a spatio-temporal plan for telotroch differentiation, apical–basal cell polarity and excystment in the ciliate *Vorticella*. In identifying the papilla as a positional constraint and the CV as an organelle generating cell polarity in a single-cell system, we provide new and additional evidence that apical–basal cell polarity is an ancestral trait conserved among prokaryotic and eukaryotic organisms. Future studies in cell biology will be required to understand how the papilla is formed during encystment, which cellular components are locally recruited to determine the site of oral cavity and apical cilia array formation, and which CV function(s) (water exclusion, ion homeostasis, signalling molecule driver) controls the cell differentiation.

## Supplementary Material



## Data Availability

Supplementary material is available online [41].

## Supplement

Supplementary material is available online at https://doi.org/10.6084/m9.figshare.c.8519877.
